# Supplementary figures and images for: Reciprocal regulation among TRPV1 channels and phosphoinositide 3-kinase in response to nerve growth factor
Source: eLife. 2018 Dec 18;7:e38869. doi: 10.7554/eLife.38869 (PMC6312403; doi:10.7554/eLife.38869)

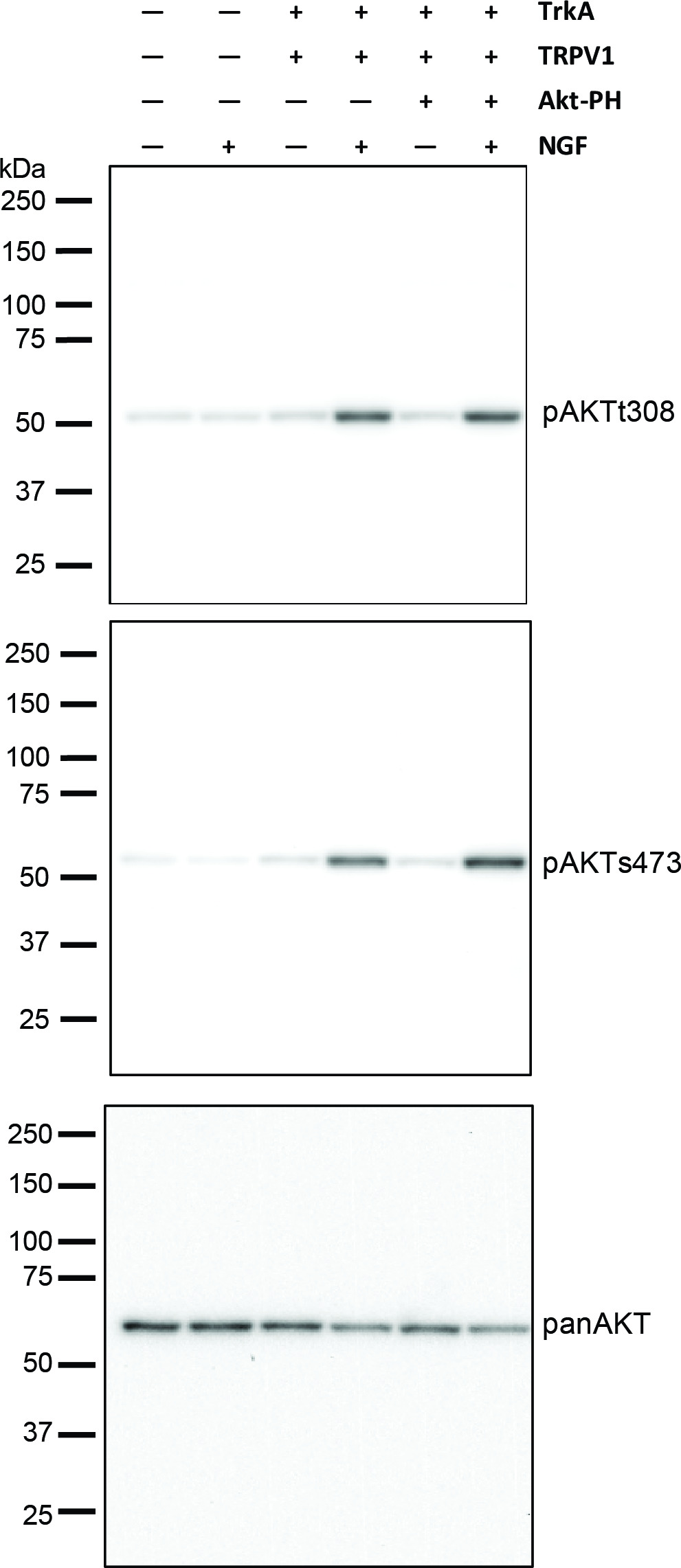

Supplement: Figure 1—figure supplement 2—source data 1. [file elife-38869-fig1-figsupp2-data1.jpg]

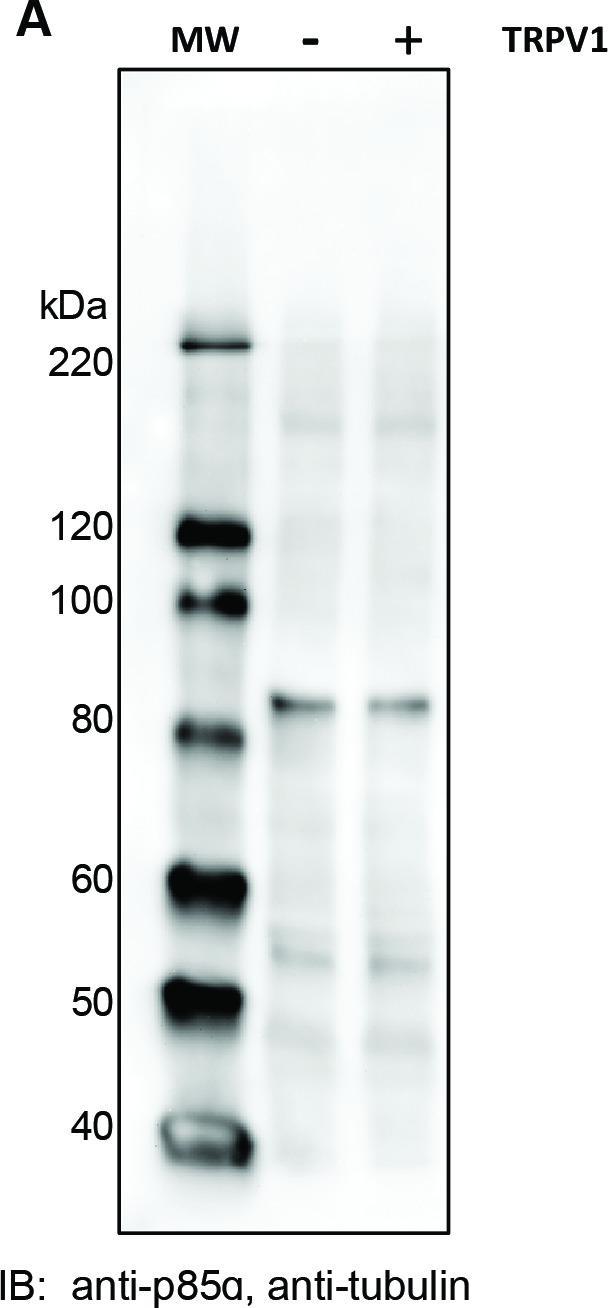

Supplement: Figure 2—figure supplement 3—source data 1. [file elife-38869-fig2-figsupp3-data1.jpg]

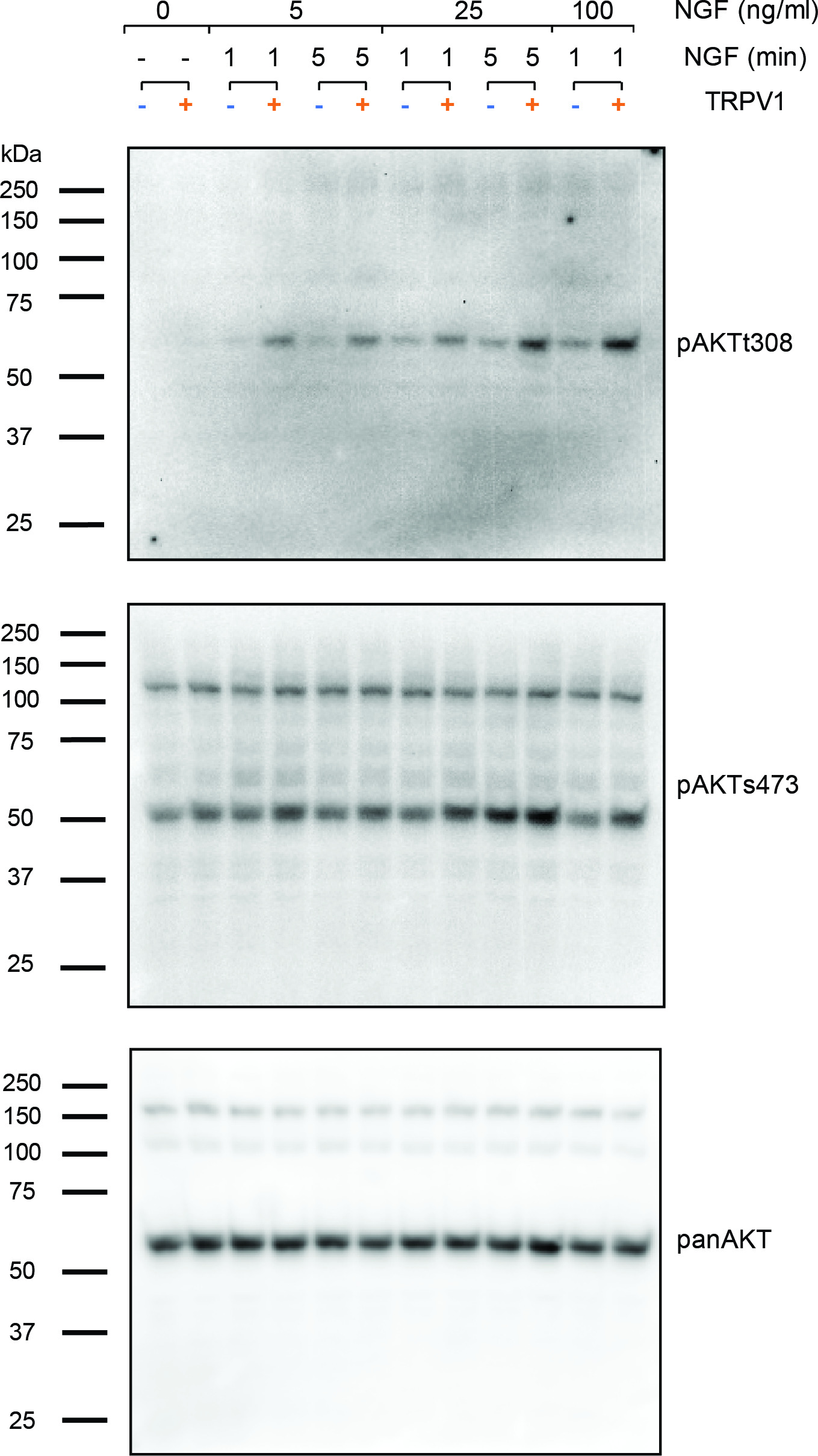

Supplement: Figure 3—source data 1. [file elife-38869-fig3-data1.jpg]
